# Phantom earthquake syndrome presenting with chronic dizziness after an earthquake: A case report

**DOI:** 10.1097/MD.0000000000042554

**Published:** 2025-05-16

**Authors:** Zeyneb İrem Yüksel Salduz, Berre Toraman

**Affiliations:** a Department of Family Medicine, Bezmialem Vakif University, Istanbul, Türkiye; b Bezmialem Vakif University, School of Medicine, Istanbul, Türkiye.

**Keywords:** case report, dizziness, earthquake, phantom earthquake syndrome, vestibular rehabilitation

## Abstract

**Rationale:**

Phantom earthquake syndrome is a rare condition seen in earthquake survivors characterized by false sense of earthquake-like motion (phantom earthquake sensations) with vegetative and motor symptoms, even though there was not any recorded earthquake by available earthquake registry. Here, we report first documented case of phantom earthquake syndrome in Turkey following the Maraş, Turkey 2023 earthquake which had presented with persistent dizziness following earthquake despite 3-month course of betahistine dihydrochloride therapy and was successfully treated with vestibular rehabilitation, lifestyle modifications and vitamin D supplementation.

**Patient concerns:**

A 44-year-old female patient was referred to our Family Medicine General Check-Up Clinic with complaints of persistent general weakness, diffuse body aches, and dizziness lasting over the previous 6 months following the Maraş, Turkey 2023 earthquake in spite of 3 months course of medical therapy.

**Diagnoses:**

The patient’s dizziness was independent of recorded aftershocks and was characterized by sensations of ground movement and earthquake-like vibrations, resulting in functional impairment due to anxiety. Comprehensive physical examination, including neurological, ophthalmological, and otoscopic evaluations, revealed no abnormalities. Laboratory studies indicated marginally elevated LDL cholesterol and triglycerides, along with slightly reduced vitamin D and thyroid-stimulating hormone levels. Cranial and cervical MRI, as well as carotid artery Doppler ultrasonography, yielded normal findings. These findings align with phantom earthquake syndrome.

**Interventions:**

Initial management by a neurologist with a 3-month course of betahistine dihydrochloride (2 × 24 mg) did not improve our patient’s symptoms. Upon the fact the patient’s earthquake experience which the patient mentioned for the first time on further history-taking along with her laboratory and imaging findings, betahistine dihydrochloride was discontinued and a treatment plan which is focusing on symptomatic relief with exercise and vestibular rehabilitation, along with vitamin D supplementation was initiated.

**Outcomes:**

After 1 month, the patient’s symptoms subsided and her biochemical and hematological parameters were within normal ranges with exercise and vestibular rehabilitation, along with vitamin D supplementation.

**Lessons:**

This case contributes to diagnose phantom earthquake syndrome as a psychosomatic response to disaster which highlights the significance of further history-taking and non-pharmacological treatments like exercise and vestibular rehabilitation for alleviating symptoms.

## 1. Introduction

On February 6, 2023, a catastrophic earthquake with a magnitude of 7.8 on the Richter scale struck 11 provinces in southeastern Turkey. Approximately 9 hours later, a second earthquake, measuring 7.7, exacerbated the devastation while search and rescue operations were still underway. Collectively, these earthquakes impacted around 15 million individuals, led to the collapse of approximately 20,000 buildings, caused structural damage to over 200,000 buildings, and resulted in more than 50,000 fatalities.^[1]^

In addition to immediate and profound structural destruction, earthquake survivors also face significant physiological, anatomical, and psychological challenges. Among these, disturbances in the vestibular system that result in balance disorders and “phantom earthquake sensations” are particularly notable. Phantom earthquake sensations refer to subjective experiences of dizziness and perceived shaking without external motion. Research indicates that psychological responses, especially heightened post-trauma, are key contributors to these sensations. The integration of reported vestibular symptoms from earthquake survivors has facilitated an operational model for understanding phantom earthquake sensations, as detailed in Table 1.^[2–4]^

**Table 1:** Model for phantom earthquake syndrome.

No recorded earthquake by available earthquake registry/detection application
False sense of earthquake-like motion (ground shaking/trembling/vibrating; sense of moving/swinging objects)
Motor and vegetative symptoms: vertigo, dizziness, nausea, motor instability, falling (“motion sickness-like” condition without motion)
Psychological distress: anxiety, insomnia, panicking, worry about losing one’s mind after false earthquake, confusion, preoccupation
Behavioral change that interferes with expected functioning: intense startle response (trigger: any environmental noise), constant alertness to earthquake cues (checking earthquake detectors), changed daily routine (avoidance of inhouse activities).

The disturbance in balance induced by the earthquake can significantly disrupt individuals’ daily lives, impairing essential functions such as walking, coordination, and vision. Consequently, a range of treatment approaches such as physiotherapy, pharmacotherapy, and psychotherapy are implemented to address earthquake-induced vestibular and balance disorders.^[5]^

Herein, we present a case of phantom earthquake syndrome in a patient with chronic dizziness which is unresponsive to pharmacotherapy. As a second case reported for phantom earthquake syndrome after a pilot study related with Zagreb and Banovina earthquake in 2020, our goal is to improve our understanding of phantom earthquake syndrome and draw attention to vestibular rehabilitation and lifestyle modifications may be more effective in comparison to pharmacotherapy.^[3]^

## 2. Case report

A 44-year-old female patient presented to the Family Medicine General Check-Up Clinic at Bezmialem Vakif University, with complaints of persistent general weakness, diffuse body aches, and dizziness lasting over the previous 6 months. The patient reported no chronic diseases, regular medication use, or recent history of head trauma. She had been using oral contraceptives for the last 3 months and had a surgical history of cholecystectomy and 3 cesarean sections. Her physical examination was unremarkable.

On further history-taking, it emerged that the patient had resided in Maraş, the epicenter of the February 6, 2023, earthquake. She reported that her dizziness commenced after the earthquake, characterized by sensations of ground shifting and episodic feelings resembling an earthquake. Additionally, she experienced intermittent anxiety, worry, and a persistent fear of an imminent earthquake. Furthermore, the patient reported that she had undergone a psychological evaluation following the earthquake, primarily due to anxiety. As stated by the patient, the anxiety she experienced did not significantly interfere with daily functioning; therefore, the psychiatrist did not establish a diagnosis of post-traumatic stress disorder (PTSD) or generalized anxiety disorder, and no pharmacological or psychotherapeutic intervention was initiated at that time. Previous management by a neurologist included a 3-month course of betahistine dihydrochloride (2 × 24 mg), which provided no symptom relief.

A detailed neurological assessment demonstrated normal consciousness and orientation, with intact motor and sensory functions, normal muscle strength, reflexes, and sensory responses. Posture and gait were within normal limits. Ophthalmological examination confirmed full visual acuity, normal eye movements, fundus appearance, and equal pupillary reflexes. Otoscopic examination showed normal external and middle ear canals and a healthy tympanic membrane. Systemic examination was unremarkable, with a temperature of 36.7°C and blood pressure of 118/75 mm Hg.

Routine laboratory tests revealed mildly elevated LDL cholesterol (131.8 mg/dL; normal < 100 mg/dL) and triglyceride levels (158 mg/dL; normal < 150 mg/dL). Vitamin D levels were slightly low (18.7 µg/L; normal 20–70 µg/L), as was thyroid-stimulating hormone (0.47 mIU/L; normal 0.55–4.78 mIU/L). A review of prior assessments, including neurology, neurosurgery, and otolaryngology evaluations, showed normal findings on cranial and cervical MRI and carotid artery Doppler ultrasonography.

A management plan was initiated, focusing on symptomatic treatment with exercise and vestibular rehabilitation, along with vitamin D supplementation. Vestibular rehabilitation exercise plan as described in Table 2 based on treatment objectives has been performed by patient 4 to 5 times daily for a total of 20 to 40 minutes/day for gaze stability, plus 20 minutes/day of balance and gait exercises.^[6]^ Each move has been performed at least twice every day, with 5 repeats of each and increasing to 10 repetitions.^[6]^

**Table 2:** Vestibular rehabilitation exercises based on treatment objectives.

1) Enhancing gaze stability
1.1 Head turns: Rotates the head side to side horizontally with gaze fixed on a stationary target. Do the same exercise with vertical head turns.
1.2 Head-trunk turns: Rotates the head and trunk together (en block) horizontally with gaze fixed on the thumb while the arm moving together with the trunk.
1.3 Head turns while walking: While walking in a straight line, the patient rotates the head horizontally to the left and right with gaze fixes on a stationary target. Do the same exercise with vertical head turns.
2) Enhancing head movements
2.1 Saccade: Keeps the head still and moves only the eyes. Imagine horizontally placed 2 targets close enough together that while looking directly at 1. Look at 1 target and quickly looks at the other target, without moving the head. These movements are repeated several times.
2.2 Pursuit: Keep the head still and moves only the eyes. Extends 1 arm forward and make the thumb (target) up and turn the arm side to side while focusing on the thumb.
2.3 Saccade and vestibulo-ocular reflex: Horizontally placed 2 targets are imagined. For example, 2 arms are extended forward with 2 thumbs (target) up. Look at a target, being sure that the head is lined up with the target. Then, look at the other target and turn the head slowly to the target. Repeat in the opposite direction. Repeat both directions several times.
2.4 Imagery pursuit (remembered target exercise): Look directly at a target, being sure that the head is lined up with the target. Close the eyes, and the head is slowly turned away from the target while imagining that one is still looking at the target. Then, open the eyes and whether the target is kept in focus is checked. If not, adjust the gaze on the target. Repeat in the opposite direction. It should be accurate as possible. Repeat both directions several times.
3) Enhancing postural stability
3.1 Stand on one leg. Stay for 15 s. Switch to the other leg.
3.2 Standing with the feet heel-to-toe with both arms extended. Stay for 15 s. Switch to the other leg.
3.3 Sway back and forth. Locate the patient behind a chair and before a wall. This prevents the patient from falling. The patient starts with bending low and move the center of body backward with the toes up. Next is bending backward and move the center of body forward with the heels up. Repeat 10 times.
3.4 March in place.
4) Decreasing vertigo
Stand with 1 arm elevated over the head, with the eyes looking at the elevated hand. Bend over and low the arm diagonally with the eyes continuously looking at the hand until the hand arrives at the opposite foot. Repeat 10 times.
5) Exercises for improving activities of daily living
5.1 Gait with sharp or wide turns to the right and left.
5.2 Go from a seated to a standing position, then return to sitting.

Upon reevaluation 1 month later in the Family Medicine Department, the patient’s biochemical and hematological parameters were within normal ranges. Notably, her symptoms of dizziness and earthquake-like sensations had markedly decreased. Written and verbal informed consent was obtained from our patient to evaluate and publish of her data in this study.

## 3. Discussion

Dizziness and vestibular symptoms have been reported across studies examining the aftermath of major earthquakes. Following the 2015 Nepal earthquakes, survivors experienced an increase in dizziness and balance disruptions.^[7]^ Similarly, early-onset dizziness and balance issues were documented after the 2020 Elaziğ earthquake in Turkey (magnitude 6.8).^[8]^ In the same year, survivors of the Zagreb earthquake (magnitude 5.5) described sensations of “phantom earthquakes.”^[4]^ Based on our literature review, this case appears to be the first reported instance in Turkey linking chronic dizziness with phantom earthquake sensation. Furthermore, while the data in the pilot study concerning the Zagreb and Banovina earthquake were derived from patients who contacted the call center for psychological support following the event, the fact that the data presented in this case report were collected through face-to-face interviews with the patient enhances the case’s significance within the context of the existing literature.^[4]^

Whether phantom earthquake sensation should be classified within the clinical spectrum of acute or PTSD in earthquake survivors or as a unique syndrome has yet to be determined. Although there is a psychological association with PTSD, phantom earthquake sensation is characterized by pronounced vegetative and motor symptoms, distinguishing it from typical PTSD presentations.^[4]^

The phantom earthquake sensation may represent a perceptual distortion, akin to an illusion or hallucination, potentially resulting from interactions between traumatic memories and abnormal sensory processing in the brain. Studies on similar perceptual phenomena point to hallucinations or illusions with proprioceptive or kinesthetic elements, where information about body position and movement is derived from multiple integrated sensory inputs rather than a single perception. In certain cases, motor regions of the brain can create conscious sensations of body posture and movement without direct sensory stimuli. The brain’s perceptual organization plays a central role in assigning importance to and integrating sensory inputs, allowing for a cohesive perception of reality through cognitive control mechanisms in cortical structures.^[9]^

Psychological responses especially intense post-trauma is considered as a key contributor to phantom earthquake sensations.^[5]^ In our case, the patient had experienced a traumatic event. Psychological stress caused by earthquake can lead to alterations in fronto-limbic-striatal network involved in perception and integration of sensory inputs, including vestibular and proprioceptive information.^[5,10,11]^ This mechanism may responsible from generation of “phantom sensations” in phantom earthquake syndrome by generating repetitive false motion perceptions without external stimuli.^[10,11]^ However, how traumatic events such as earthquakes and the resulting anxiety may impact brain function to generate repetitive false motion perceptions remains unclear, highlighting a need for further research on the neurobiological effects of severe anxiety on distinguishing real vs false stimuli.

Mal de débarquement syndrome (MdDS), or “disembarkation sickness,” presents a related pathology, characterized by prolonged dizziness and sensations of movement following extended sea travel or long-haul flights. Unlike phantom earthquake sensation, MdDS is typically chronic and often presents with specific neuroimaging findings.^[12]^

Vitamin D is a fat-soluble vitamin mainly responsible for calcium homeostasis by exerting its effects through a vitamin D receptor (VDR), which is expressed by all nucleated cells in human body.^[13]^ Beside the effects on calcium homeostasis, recent studies indicate low serum vitamin D levels may be correlated with increased incidence and recurrence of benign paroxysmal positional vertigo (BPPV), associated with otolithic dysfunction and degeneration of vestibular neurons characterized by abnormal vestibular-evoked myogenic potentials results.^[13–15]^ The effect of vitamin D insufficiency in BPPV could be implied with increased otolith dysfunction and abnormal impulses generated by hair cells due to inadequate calcium concentrations in inner ear and loss of ganglion cells in vestibular neurons.^[15,16]^ In our case, the patient has slightly decreased vitamin D levels (18.7 µg/L; normal 20–70 µg/L), which is corrected after supplementation therapy. Along with vestibular rehabilitation, improvement of phantom earthquake sensation may be augmented by potential effects of vitamin D on hair cells, vestibular neurons and otoliths.^[15]^ Although the impact of vitamin D on phantom earthquake syndrome warrants further investigation, limited population of patients with phantom earthquake syndrome may limit further studies.

Turkey is considered as one of the most susceptible area to earthquakes all around the world due to its seismically active tectonic settings.^[17]^ Under that context, devastating nature of frequent earthquake history in Turkey exerts itself in Turkish culture as folk poetry and memorates representing the outlook and coping mechanisms of Turkish community.^[18]^ According to study conducted by Çevirme, Turkish earthquake culture mostly involves fatalistic approach, characterized by considering the cause of the earthquake is God or destiny as a coping mechanism through poetry and memorates.^[18]^ In our case, the fatalistic approach of the Turkish community may have prevented our patient to have severe anxiety or PTSD to the extent that may affect the quality of life and facilitated easier acceptance of negative consequences.

In this case, the patient’s dizziness was unrelated to recorded aftershocks and was accompanied by reduced anxiety and improved social functioning, consistent with a diagnosis of phantom earthquake sensation, despite the fact that the complaints associated with the diagnosis of “phantom earthquake syndrome” were subjective in nature. Pharmacological treatment yielded no improvement; however, vestibular rehabilitation and lifestyle modifications led to symptom resolution. This case underscores the importance of preventive medicine approaches to address post-earthquake vestibular disturbances and associated psychological effects.

## 4. Conclusion

The phantom earthquake sensation, defined as the false perception of earthquake-like movement, may be linked to underlying brain mechanisms influenced by trauma and anxiety. This case underscores the significance of employing a biopsychosocial approach and conducting detailed history-taking during patient evaluations. The critical role of preventive medicine, lifestyle modifications, and precautionary interventions should not be overlooked, as these strategies are vital for facilitating the reintegration of earthquake survivors into their daily lives. Effective management of dizziness related to earthquake-induced psychological stress necessitates an interdisciplinary assessment, which should include a comprehensive evaluation of underlying disorders along with the implementation of appropriate counseling and therapeutic approaches.

## Acknowledgments

The authors would like to acknowledge the use of ChatGPT, an AI language model developed by OpenAI, for assisting with the rephrasing and refinement of certain sections of the manuscript. ChatGPT was used solely as a tool to enhance clarity and improve the language of the text. The authors take full responsibility for the content, analysis, and conclusions of the case report, and no clinical or medical decisions were influenced by the AI tool.

## Author contributions

**Supervision:** Zeyneb İrem Yüksel Salduz.

**Writing – original draft:** Zeyneb İrem Yüksel Salduz, Berre Toraman.

**Writing – review & editing:** Zeyneb İrem Yüksel Salduz, Berre Toraman.
